# Dehydroepiandrosterone exacerbates nigericin-induced abnormal autophagy and pyroptosis via GPER activation in LPS-primed macrophages

**DOI:** 10.1038/s41419-022-04841-6

**Published:** 2022-04-19

**Authors:** Ji Cao, Longlong Li, Yao Yao, Yuxiao Xing, Haitian Ma

**Affiliations:** 1 Key Laboratory of Animal Physiology and Biochemistry, College of Veterinary Medicine, Nanjing Agricultural University, Nanjing, 210095 PR China; 2 MOE Joint International Research Laboratory of Animal Health and Food Safety, College of Veterinary Medicine, Nanjing Agricultural University, Nanjing, 210095 PR China

**Keywords:** Cell death and immune response, Immune cell death

## Abstract

As a widely acknowledged FDA-approved dietary supplement or over-the-counter medicines, dehydroepiandrosterone (DHEA) exerts anti-inflammatory and immunomodulatory function. Pyroptosis is an important form of programmed cell death (PCD), and which acts a key role in the body’s anti-infection and inflammatory responses. But the effects and mechanisms of DHEA on pyroptosis remain unclear. Here, we found that DHEA inhibited the NLRP3 inflammasome components expression by blocking inflammatory signals in lipopolysaccharide (LPS)-primed macrophages, and prevented the bacterial toxin nigericin (Nig)-induced NLRP3 inflammasome assembly. However, DHEA exacerbated NLRP3-independent cell death in Nig-treated inflammatory macrophages. During this process, DHEA induced the abnormal autophagy, which reflected as the blocking of autophagic flux and the accumulation of autophagy receptor p62 (SQSTM1) protein. In addition, DHEA caused a burst of reactive oxygen species (ROS) and activated extracellular signal-regulated kinase (ERK) phosphorylation in LPS plus Nig-stimulated macrophages but not in LPS-treated macrophages. Mechanistically, the present study certified that the activation of G protein-coupled estrogen receptor (GPER) signal mediated the cell death induced by DHEA in Nig-stimulated inflammatory macrophages, as GPER specific inhibitor G15 alleviated the abnormal autophagy and ultimately prevented the gasdermin D (GSDMD)-mediated pyroptosis induced by DHEA. Collectively, DHEA can exacerbate Nig-induced abnormal autophagy and pyroptosis via activation of GPER in LPS-primed macrophages, which prompts us the potential application value of DHEA in anti-infection or anti-tumor immunity.

## Introduction

As an important component of innate immune response, inflammasome can respond to a variety of stimuli, including pathogen invasion, danger signals generated by host cells, and environmental stimuli [1]. The NOD-like receptor family, pyrin domain containing 3 (NLRP3) inflammasome is one of the most widely studied inflammasomes, and that consist of the NLRP3, adaptor protein apoptosis-associated speck-like protein (ASC) and caspase-1 [2], and which acts an important role in immune-related diseases including sepsis and other infectious diseases [3, 4]. The activation process of canonical NLRP3 inflammasome includes two steps: In signal 1 (priming), intracellular nuclear factor kappaB (NF-κB) pathway is activated to induce the expression of NLRP3, interleukin-1β precursor (pro-IL-1β) and caspase-1 precursor (pro-caspase-1); In signal 2 (activating), NLRP3 can be activated by multiple stimuli such as nigericin (Nig) or adenosine triphosphate (ATP) [5, 6]. Activated NLRP3 inflammasome can result in caspase-1-mediated maturation of several inflammatory cytokines (e.g. IL-1β and IL-18) and induce a gasdermin-mediated programmed cell death (called pyroptosis) [7, 8]. Pyroptosis is characterized as the continuous expansion of cells until the cell membrane ruptures, that finally results in the release of cell contents and activates a strong inflammatory response [9–11]. Up to now, the mechanism by which gasdermin D (GSDMD) induces pyroptosis is relatively clear. Inflammatory caspases can cleave GSDMD and release its active fragment GSDMD-NT. The GSDMD-NT can punch holes in the cell membranes of bacteria that are infecting host cells, thereby killing these bacteria. Meanwhile, it can also perforate the cell membranes of host cells and arise the pyroptosis, thereby killing host cells, releasing bacteria and immune warning signs [11, 12]. It is noteworthy that pyroptosis also lead to the excessive release of inflammatory factors in inflammatory disease including sepsis [13, 14]. Accordingly, proper regulation of pyroptosis is critical for the treatment of systemic inflammation.

Dehydroepiandrosterone (DHEA, 3β-hydroxy-5-androstene-17-one) is an important cholesterol-derived intermediate that function both as the major steroid hormone with systemic endocrine function in humans, and as a food supplement or drug approved by FDA [15–18]. Our and other researchers found that DHEA has anti-inflammation and anti-oxidant activities in several mouse inflammatory model [19–21], which implied that it can be used as a potential anti-inflammatory supplemental agent. G protein-coupled estrogen receptor (GPER) has been shown to mediate rapid non-genomic estrogenic effects of estrogenic compounds (such as DHEA) [22]. Our recent and other researches also found that DHEA can activate GPER to perform its biological functions [20, 23, 24]. In addition, it has been reported that activated GPER can decrease the toll-like receptor 4 (TLR4)-mediated inflammation in murine macrophages and microglia [25, 26]; and GPER agonist can inhibit the activation of NLRP3 inflammasomes [27]. However, whether DHEA can regulate the NLRP3 inflammasome activation and then affect pyroptosis, and whether these actions are mediated by GPER is largely unclear.

Here, we specifically investigated the effects and mechanisms of DHEA on the NLRP3 inflammasome activation and pyroptosis in macrophages, and found that DHEA can inhibit the inflammatory signal and NLRP3 inflammasome activation. However, DHEA induces abnormal autophagy and exacerbates pyroptosis in Nig-treated inflammatory macrophages via GPER activation. In this study, we confirmed that although DHEA exerts anti-inflammatory activity, it can also aggravate cell death in Nig-treated inflammatory macrophages. The regulation of pyroptosis by DHEA may contribute to its application in anti-infection or anti-tumor immunity.

## Results

### DHEA inhibits the activation of inflammatory signal and NLRP3 inflammasome in macrophages

**Fig. 1:**
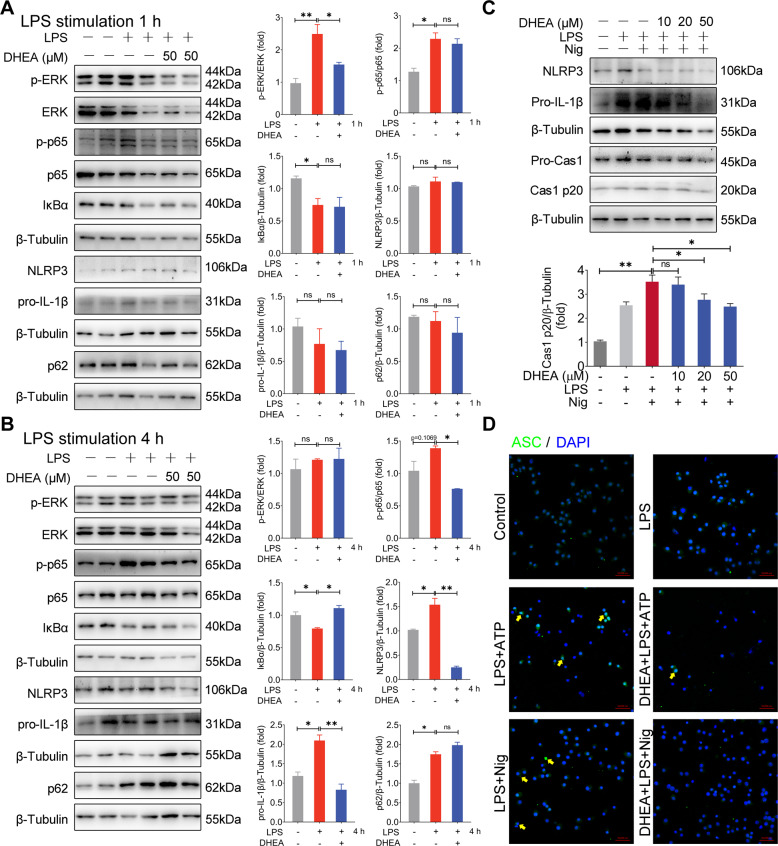
DHEA inhibits the activation of inflammatory signal and NLRP3 inflammasome in macrophages. **A**, **B** J774A.1 cells were pre-treated with DHEA (50 μM) for 1 h, then the cells were stimulated with 100 ng/mL LPS for 1 or 4 h. After that, the p-ERK/ERK, p-p65/p65, IκBα, NLRP3, pro-IL-1β, and p62 protein expression levels were measured by western blotting and quantified by Image J software. **C** J774A.1 cells were pre-treated with different doses of DHEA (0, 10, 20, 50 μM) for 1 h and primed with 100 ng/mL LPS for 4 h; then stimulated with NLRP3 activators nigericin (10 μM) for 1 h. The NLRP3, pro-IL-1β, pro-Cas1, and Cas1 p20 protein levels were measured by western blotting. **D** After indicated treatments, the ASC speck formation was analyzed by immunofluorescence, scale bar = 50 μm. Data are presented as means ± SEM (*n* = 3). *P < 0.05, **P < 0.01, compared with the respective control.

No differences were observed on cell viability of murine macrophages cell line J774A.1 cells treatment with the concentration of 10-50 μM DHEA for 12 h (sFig. 1A), which indicated that there is no cytotoxicity in J774A.1 cells as exposed to up to the concentration of 50 μM DHEA. Subsequently, we analyzed the effects of DHEA (50 μM) on the inflammatory signal and the expression of NLRP3 components in LPS-stimulated macrophages. As shown in Fig. 1A, LPS stimulation (1 h) significantly increased the pro-inflammatory factors of ERK, p65 phosphorylation levels and decreased the IκBα protein expression level, while DHEA treatment significantly inhibited p-ERK expression level. When LPS stimulation for 4 h, DHEA obviously inhibited the p-p65 but enhanced IκBα protein levels (Fig. 1B). Meanwhile, the protein levels of NLRP3 and pro-IL-1β were significantly increased in J774A.1 cells after LPS treatment for 4 h, and DHEA reduced their expression levels (Fig. 1B). In addition, we also found that LPS alone treatment increased the level of autophagy receptor protein p62 (also called SQSTM1), the downstream factor of NF-κB, which is consistent with the previous reports [28], while DHEA has no significant effect on p62 protein level (Fig. 1B). To further explore the effect of DHEA on NLRP3 activation, we established an NLRP3 inflammasome activation model using the murine macrophages cell line J774A.1, which is widely be used in investigation the mechanism of inflammasome activation [29]. As shown in Fig. 1C, DHEA decreased the NLRP3, pro-IL-1β and pro-caspase-1 protein levels with a dose-dependent manner in LPS plus nigericin (Nig) treated J774A.1 cells (Fig. 1C); and DHEA also inhibited the LPS + Nig-induced caspase-1 (Cas1) mature p20 form expression level in J774A.1 cells (Fig. 1C). Besides, the confocal imaging further showed that DHEA blocked the ASC speck formation in LPS + Nig or LPS + ATP-treated J774A.1 cells (Fig. 1D). These data demonstrated that DHEA can inhibit the activation of inflammatory signal pathway (ERK, NF-κB) and NLRP3 inflammasome in macrophages.

### DHEA promotes Nig-induced pyroptosis in LPS-primed macrophages

**Fig. 2:**
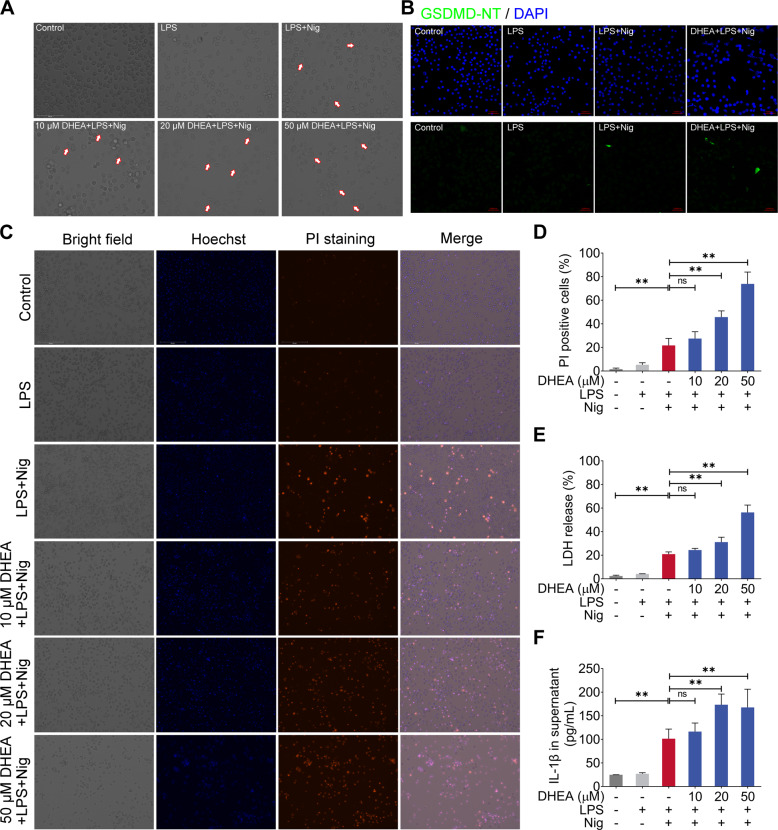
DHEA promotes Nig-induced pyroptosis in LPS-primed macrophages. **A** Cells were pre-treated with different doses of DHEA (0, 10, 20, 50 μM) for 1 h and primed with LPS for 4 h; then stimulated with nigericin (Nig) for 1 h. Cell morphology was observed by light microscopy, the red arrows represent the cells that burst due to pyroptosis, scale bar = 100 μm. **B** The GSDMD-NT protein levels were analyzed by immunofluorescence, scale bar = 50 μm. **C**, **D** PI-positive dead cells in 5 randomly selected fluorescence microscope images were counted by Image J software, scale bar = 200 μm. **E** The cell death was measured by detecting the lactate dehydrogenase (LDH) release (%) in cell culture supernatant. **F** IL-1β content in cell culture supernatant. Data are presented as means ± SEM (*n* = 4). *P < 0.05, **P < 0.01, compared with the respective control.

Next, we observed the cells morphology in LPS + Nig-stimulated macrophages treated with different concentrations of DHEA under the microscope. Interestingly, DHEA obviously increased the proportion with typical pyroptosis morphological characteristics in macrophages treated with LPS + Nig in a dose-dependent manner; especially 20 and 50 μM DHEA treatment obviously caused the cell membrane rupture in macrophages (Fig. 2A). According to reports, pyroptosis can be mediated by the effector molecules GSDMD [11]. In this study, the immunofluorescence analysis showed that 50 μM DHEA significantly increased expression of GSDMD-NT, suggested that DHEA promoted GSDMD-mediated pyroptosis (Fig. 2B). To further verify whether DHEA could promote the pyroptosis in LPS + Nig-treated macrophages, the propidium iodide (PI)-staining and lactate dehydrogenase (LDH) release were employed to indicate the cell death, and the ELISA kit was used to detect the IL-1β level in the cell supernatant. Results showed that DHEA treatment significantly increased the proportion of PI-positive cells and the release of LDH in a dose-dependent manner (Fig. 2C–E). Besides, 20 and 50 μM DHEA treatment obviously increased IL-1β content in the cell culture supernatant (Fig. 2F). These results strongly implied that DHEA promotes the Nig-induced pyroptosis in LPS-primed macrophages. However, it is worth noting that although NLRP3 inhibitor MCC950 and NF-κB inhibitor BAY11-7082 can inhibit the cell death caused by LPS + Nig stimulation in macrophages, while NLRP3 inhibitor MCC950 cannot reverse the cell death phenomenon exacerbated by DHEA (sFig. 1B and C). In addition, we also found that DHEA treatment significantly improved the tumor necrosis factor-α (TNF-α) concentration in cell culture supernatant (sFig. 1D). These results confirmed that DHEA can promote GSDMD-induced pyroptosis in Nig-stimulated inflammatory macrophages, and this effect presents an independent of NLRP3 inflammasome activation.

### DHEA induces abnormal autophagy and excessive release of ROS in Nig-treated inflammatory macrophages

**Fig. 3:**
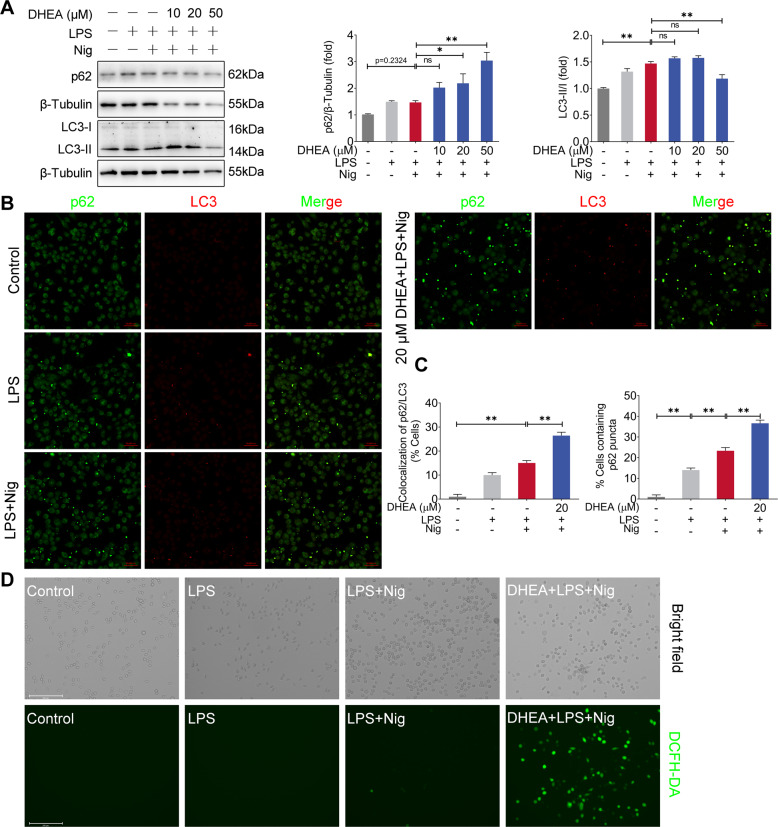
DHEA induces abnormal autophagy and excessive release of ROS in Nig-treated inflammatory macrophages. **A** After indicated treatments, the p62 and LC3 protein levels were analyzed by western blotting and quantified by Image J software. **B**, **C** The formation of p62/LC3 puncta was analyzed by immunofluorescence, scale bar = 50 μm. **D** The intracellular ROS levels were detected using DCFH-DA, scale bar = 200 μm. Data are presented as means ± SEM (*n* = 3). *P < 0.05, **P < 0.01, compared with the respective control.

Autophagy is mainly an important cell protection process and is closely related to pyroptosis [30–32]. Thus, we tested the effects of DHEA on autophagy in LPS + Nig-stimulated macrophages. As shown in Fig. 3A, LPS + Nig treatment significantly increased the expression level of autophagy marker LC3 II, and it also up-regulates the expression of p62 protein to some extent. We found that 20 and 50 μM DHEA treatment further increased the p62 protein level. However, 50 μM DHEA inhibited the expression of LC3 II (Fig. 3A). Furthermore, it was found that 20 μM DHEA induced the colocalization of p62/LC3 puncta and caused more p62 puncta accumulation in macrophages by immunofluorescence (Fig. 3B, C), suggested that DHEA may induce a blocked autophagic flux. These data demonstrated that DHEA has an inhibitory effect on autophagy in the Nig-stimulated inflammatory macrophages.

Based on the above results, we subsequently used mCherry-GFP-LC3B adenovirus reporter (Beyotime) to further examine the effect of DHEA on autophagic flux. sFig. 2A revealed that the number of both yellow (autophagosome) and red puncta (autolysosome) were increased in LPS + Nig-treated macrophages, while DHEA treatment obviously decreased the number of red puncta, implied that DHEA inhibits the formation of autolysosome and induced abnormal autophagy in the LPS + Nig-treated macrophages. This result also explains the accumulation of p62 puncta induced by DHEA (Fig. 3B, C). Accompanied with abnormal autophagy, we found that DHEA caused a burst of reactive oxygen species (ROS) in LPS + Nig-stimulated macrophages (Fig. 3D) but not in LPS-treated macrophages (Data not shown). Taken together, DHEA induced abnormal autophagy and excessive release of ROS, and which eventually promotes the pyroptosis in Nig-stimulated inflammatory macrophages.

### DHEA activates GPER and AMPK/mTOR signal in inflammatory macrophages

**Fig. 4:**
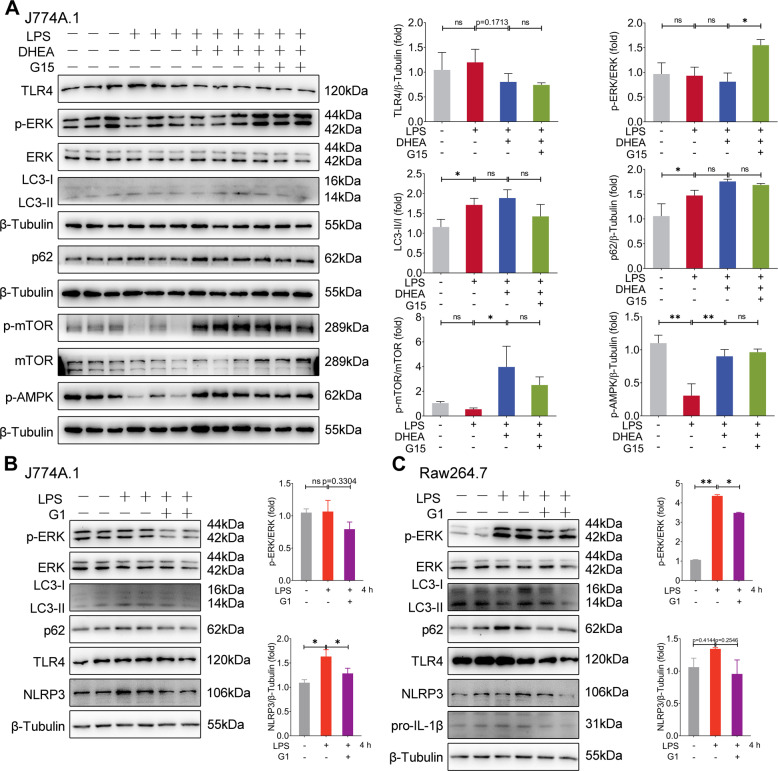
DHEA activates GPER and AMPK/mTOR signal in inflammatory macrophages. **A** J774A.1 cells were pre-treated with DHEA (50 μM) in the presence or absence of the GPER inhibitor G15 (1 μM) for 1 h, then stimulated with LPS for 4 h. The TLR4, p-ERK/ERK, LC3, p62, p-mTOR, and p-AMPK protein expression levels were measured by western blotting and quantified by Image J software. **B**, **C** J774A.1 and RAW264.7 cells were pre-treated with G1 (1 μM) for 1 h, then stimulated with LPS for 4 h, and the indicated protein expression levels were measured by western blotting and quantified by Image J software. Data are presented as means ± SEM (*n* = 3 or 4). *P < 0.05, **P < 0.01, compared with the respective control.

We further investigated the effect of DHEA on GPER and its downstream signaling in LPS-treated macrophages. As shown in Fig. 4A, the GPER specific inhibitor G15 significantly increased ERK phosphorylation level (but had no effect on TLR4 protein level) than that of the DHEA-treated group, which implied that the inhibition effect of DHEA on the pro-inflammatory ERK signal is mediated by GPER in LPS-treated macrophages. In addition, compared with the LPS-treated group, there is no significant difference on expression levels of LC3 II and p62 in cells treated with DHEA or G15 + DHEA (Fig. 4A), which implied that DHEA or GPER signal does not induce abnormal autophagy in LPS-treated macrophages. Meanwhile, we found that DHEA caused the simultaneous activation of AMPK and mTOR in LPS-stimulated macrophages, while G15 does not affect this effect, which indicated that the ability of DHEA to activate AMPK/mTOR is independent of GPER activation in inflammatory macrophages.

**Fig. 5:**
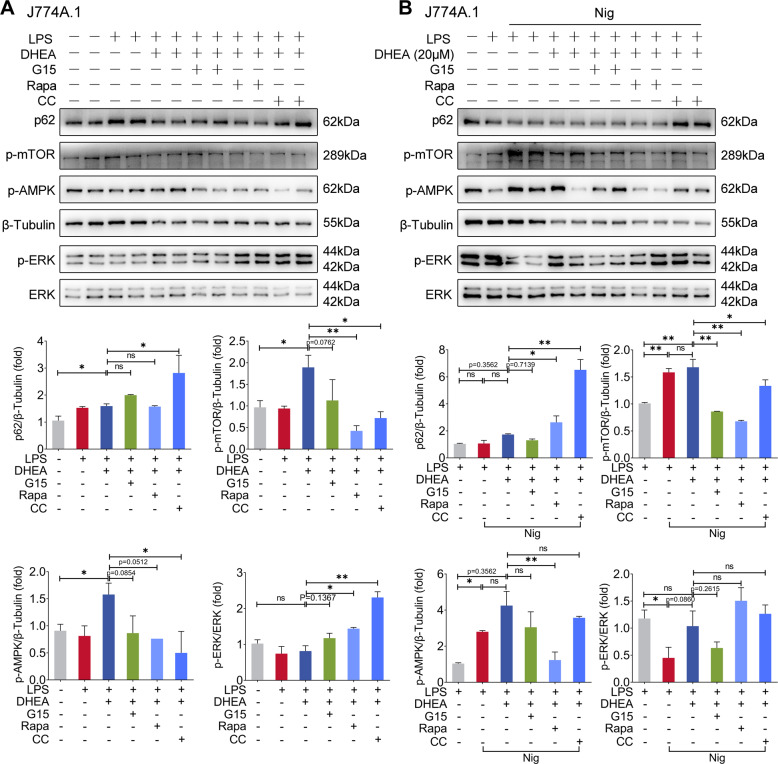
DHEA reactivates ERK signaling and induces p62 accumulation in Nig-treated inflammatory macrophages via GPER activation. **A** J774A.1 cells were pre-treated with DHEA (50 μM) in the presence or absence of the mTOR inhibitor rapamycin (Rapa) (100 nm) or AMPK inhibitor compound C (CC) (10 μM) for 1 h, then stimulated with LPS for 4 h, the indicated protein expression levels were measured by western blotting and quantified by Image J software. **B** Cells were pre-treated with DHEA (20 μM) in the presence or absence of Rapa or CC for 1 h, and primed with LPS for 4 h; then stimulated with nigericin (Nig) for 1 h, the indicated protein expression levels were measured by western blotting and quantified by Image J software. Data are presented as means ± SEM (*n* = 4). *P < 0.05, **P < 0.01, compared with the respective control.

Next, we activated GPER using the GPER agonist G1 in two macrophage cell lines (J774A.1 and RAW264.7), the results showed that G1 can also inhibit ERK activation in LPS- stimulated macrophages and reduce the NLRP3-related proteins expression levels downstream of TLR4 signal (Fig. 4B). Unlike the results of DHEA treatment that presented in Fig. 4A, G1 treatment decreased the expression levels of LC3 II and p62 to some extent (Fig. 4B), and G1 treatment failed to induce activation of AMPK/mTOR (sFig. 3A and B). Interestingly, we found that ERK inhibitor U0126 treatment reduced the expression levels of LC3 II and p62 in LPS-treated macrophages (sFig. 4A), implied that activation of ERK induces autophagy, which is consistent with previous reports [33]. Thus, we speculated that GPER activation partially inhibited ERK-induced autophagy, and the activating AMPK/mTOR signal may prevent GPER-induced autophagy inhibition. To confirm this conjecture, we used AMPK inhibitor compound C (CC) and mTOR inhibitor rapamycin (Rapa), respectively, to block the activation of AMPK/mTOR signal in LPS-treated macrophages. The results showed that pre-treated with both Rapa and CC enhanced the phosphorylation level of ERK in cells after DHEA treatment, and CC treatment also induced a significant up-regulation in p62 protein level (Fig. 5A); which implied that AMPK/mTOR also involved in the inhibition of ERK signaling, and the activation of AMPK signaling induced by DHEA contributes to the autophagic degradation of p62 protein. The above results suggested that GPER and AMPK/mTOR signal are both involved in the anti-inflammatory effect of DHEA in LPS-treated macrophages.

### DHEA reactivates ERK signaling and induces p62 accumulation in Nig-treated inflammatory macrophages via GPER activation

However, we found that the phosphorylation levels of AMPK and mTOR were significantly enhanced in LPS-primed macrophages stimulated with Nig, while DHEA could not further alter AMPK/mTOR in Nig-treated inflammatory macrophages (Fig. 5B). Correspondingly, we also found that LPS + Nig treatment obviously reduced the phosphorylation level of ERK, but had no influence in p62 level compared with LPS treatment group (Fig. 5B). However, DHEA reactivated ERK signal and increased p62 protein expression (Fig. 5B). The immunofluorescence results also confirmed that the ERK inhibitor U0126 obviously alleviated the p62 puncta formation that induced by DHEA in LPS + Nig-stimulated macrophages (sFig. 4B). These results suggested that DHEA can reactivate ERK to maintain p62 expression level. The above results in this study showed that DHEA can lead to a blocked autophagic flux, while lysosome is an important place for fusion with autophagosome to form autophagolysosome and play a role in degradation [34]. Thus, we subsequently employed the LysoTracker Red staining to analyze lysosomal function in cells, the results showed that DHEA induced significant lysosomal damage in LPS + Nig-stimulated macrophages, and which was alleviated by G15 to some extent (sFig. 5A and B). Furthermore, we found that LPS + Nig treatment can promote mTOR lysosomal translocation (lysosomes were labeled with LAMP1) by immunofluorescence (sFig. 5C), which has been shown to be an important cause of lysosomal damage [35]. DHEA treatment further exacerbated the mTOR puncta colocalized with LAMP1, while G15 can reverse this effect of DHEA (sFig. 5C), indicated that GPER mediates the effect of DHEA to exacerbate lysosomal damage, ultimately leading to the abnormal autophagy. These findings demonstrated that DHEA can reactivate ERK signal and induce p62 accumulation via GPER activation in Nig-treated inflammatory macrophages.

### DHEA exacerbates Nig-induced pyroptosis in LPS-primed macrophages via GPER activation

**Fig. 6:**
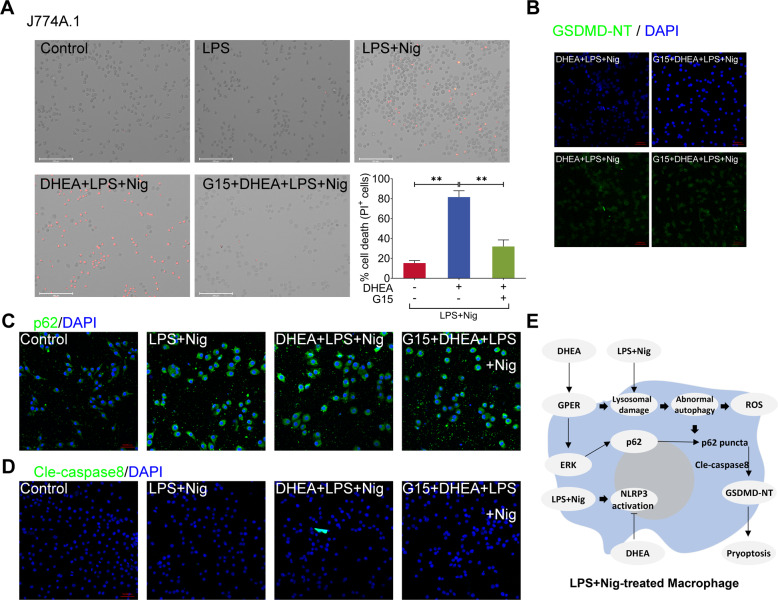
DHEA exacerbates Nig-induced pyroptosis in LPS-primed macrophages via GPER activation. **A** J774A.1 cells were pre-treated with DHEA (50 μM) in the presence or absence of the GPER inhibitor G15 (1 μM) for 1 h, then stimulated with LPS for 4 h, PI-positive dead cells in 5 randomly selected fluorescence microscope images were counted by Image J software, scale bar = 200 μm. **B** The GSDMD-NT protein levels were analyzed by immunofluorescence, scale bar = 50 μm. **C** The p62 puncta formation was analyzed by immunofluorescence, scale bar = 50 μm. **D** The cleaved caspase-8 protein levels were analyzed by immunofluorescence, scale bar = 50 μm. **E** Possible mechanism by which DHEA exacerbates cell death. Data are presented as means ± SEM (*n* = 3). **P < 0.01, compared with the respective control.

In the present study, we found that GPER inhibitor G15 obviously decreased the GSDMD-NT protein expression level and reversed Nig-induced pyroptosis in LPS-primed J774A.1 macrophages (Fig. 6A, B). Similarly, we also found that G15 treatment prevented the enhancement of DHEA-induced GSDMD-NT expression and pyroptosis in RAW264.7 macrophages (sFig. 6A and B), it should be noted that LPS + Nig cannot induce NLRP3-dependent pyroptosis due to the lack of adaptor protein ASC in RAW264.7 macrophages [36]. In addition, G15 could also reduce the p62 puncta formation in J774A.1 macrophages (Fig. 6C). Notably, DHEA obviously enhanced the cleaved caspase-8 (Cle-caspase-8) protein expression level in LPS + Nig-treated macrophages, but G15 treatment reversed this effect of DHEA (Fig. 6D). According to reports, the protein p62 was shown to activate caspase-8 [37, 38]; and activated caspase-8 can cleave GSDMD to promote cell pyroptosis [39, 40]. Thus, these results confirmed that DHEA exacerbates Nig-induced pyroptosis in LPS-primed macrophages via GPER activation, and the DHEA-caused pyroptosis may be related to p62-caspase-8-GSDMD pathway.

## Discussion

Dehydroepiandrosterone (DHEA) has long been considered as the major precursor of steroid hormone with systemic endocrine function in humans [15, 16, 41]. In recent years, the immune regulation roles of DHEA have been attracted more attention. Researches from our laboratory and others had certified that DHEA can indeed prevent inflammatory damage [19, 42, 43]. Although it is well known that pyroptosis plays critical role in the innate immune defense, but the regulation effects and mechanisms of DHEA on pyroptosis is still unclear. In the present study, we demonstrated that DHEA blocks the inflammatory signal and NLRP3 inflammasome activation. But to our surprise, we found that DHEA induces abnormal autophagy and exacerbates pyroptosis in Nig-treated inflammatory macrophages via GPER activation.

NLRP3 inflammasome activation have been involved in various inflammatory diseases such as type 2 diabetes, atherosclerosis, gout, Alzheimer’s disease, and inflammatory bowel disease (IBD) [44–46]. Our previous study found that DHEA prevents NLRP3 inflammasome activation in the intestinal epithelium and inhibits colitis in mice [20]. In the present study, we also found that DHEA inhibited the NLRP3 inflammasome components expression in LPS-treated J774A.1 cells, and this effect is associated with the inhibition of ERK and NF-κB inflammatory signaling upstream of NLRP3, these data implied that DHEA can be used as a potential anti-inflammatory drug. Consistent with the above results, DHEA also blocked the ASC speck formation in LPS + Nig or LPS + ATP-treated J774A.1 cells, as ASC speck formation is important in the assembly process of the NLRP3 inflammasome [47]. These results suggested that DHEA suppresses the NLRP3 inflammasome activation, and which mainly through inhibiting the activation of inflammatory signaling in macrophages.

GPER is a newly discovered new type of estrogen receptor that can mediate the rapid signal transmission capability for steroid hormone including DHEA [22, 24], and the increasing evidence showed that GPER can inhibit TLR4-mediated inflammatory response in macrophages [25]. As TLR4 signal participates in the priming or activating process of NLRP3 inflammasome [48], thus we speculated that DHEA may suppress the NLRP3 inflammasome activation via GPER signaling in macrophages. In this study, although we did not find significant changes in TLR4 expression by DHEA or GPER activator G1 treatment in LPS-primed macrophages, while DHEA or G1 prevented the activation of ERK and NF-κB signal. Taking the above results, our findings indicated that DHEA suppresses the inflammatory response and inflammasome activation by activating the GPER in LPS-induced macrophages.

Autophagy is mainly an important cell protection process. Through this process, cells can isolate damaged proteins, organelles such as mitochondria or pathogens in double-membrane compartments, namely autophagosomes, and then target them to lysosomes for degradation and recycling [49]. Autophagy occurs under normal physiological conditions, and its level can be up-regulated through starvation or bacterial infection [49]. Autophagy acts an important role in regulating the body’s inflammatory response and immune status [50]. Baseline autophagy weakens the release of IL-1β, and which related to the clearance of impaired mitochondria (an important factor induces the NLRP3 inflammasome activation) [51]; Autophagy has also been reported to have the effect of inhibiting pyroptosis [30, 52], but abnormal autophagy fails to clear the accumulation of ROS in cells, which may eventually induce inflammatory response and cell death [53, 54]. In the present study, we found that DHEA increased the expression level of the autophagy protein LC3 II in LPS-stimulated macrophages to some extent (indicates that autophagy levels are elevated), which was related to its apparent activation of AMPK/mTOR signaling, since AMPK/mTOR is the key molecule regulating autophagy (AMPK activation promotes autophagy, while mTOR activation inhibits autophagy) [55]. In addition, we found that pre-treated with both AMPK inhibitor compound C (CC) and mTOR inhibitor rapamycin (Rapa) enhanced the phosphorylation level of ERK in cells after DHEA treatment, which indicated that AMPK/mTOR involved in the inhibition of ERK signaling; besides, CC treatment induced a significant up-regulation in p62 protein level, which implied that the activation of AMPK signaling induced by DHEA contributes to the autophagic degradation of p62 protein in LPS-treated macrophages. However, as Nig was added to LPS-priming macrophages, the AMPK/mTOR signaling was significantly activated (at this time, LPS + Nig stimulation increased the levels of LC3 II and p62 in macrophages), while DHEA had no significant effect on AMPK/mTOR signaling pathway. It has been reported that AMPK can be activated by inflammasome inducers (adenosine triphosphate, ATP) and plays an important role in inflammasome activation [56]. This is similar to what we got in the present study. Interestingly, we found LPS + Nig stimulation caused the inhibition of ERK signal, but DHEA could reactivate ERK signal via GPER activation. Notably, activation of ERK reflects the rapid estrogenic effect of DHEA via GPER [20, 22]. Although our data showed that ERK signal can activate autophagy, we further found that DHEA treatment induced severe damage to lysosomes than that of LPS + Nig treatment, which may ultimately lead to blocking the autophagic flux in macrophages, and accompanied with the accumulation of autophagy substrate protein p62. These data demonstrated that GPER and AMPK/mTOR signal are both involved in the function (anti-inflammation, maintain normal autophagy) of DHEA in LPS-treated macrophages; but in LPS + Nig-treated macrophages, DHEA can reactivate ERK signaling and block the process of autophagy via GPER activation, and which ultimately induce p62 accumulation and ROS overproduction.

Pyroptosis is a gasdermin-mediated programmed cell death [12]. In this study, we found that DHEA promoted pyroptosis in an NLRP3-independent manner, as pretreatment with the NLRP3 inhibitor MCC950 cannot reverse DHEA-induced cell death in LPS + Nig-stimulated macrophages. Interestingly, the GPER inhibitor G15 prevented DHEA-induced the increasing of GSDMD-NT expression level and subsequent pyroptosis, which implied that DHEA-induced pyroptosis is dependent on GPER activation. In fact, GPER mediated the abnormal autophagy induced by DHEA in LPS + Nig-stimulated macrophages, which led to the p62 puncta aggregation and caspase-8 activation, ultimately induced the production of GSDMD-NT and caused pyroptosis. Taken together, we thought that DHEA-induced abnormal autophagy was involved in the exacerbation of pyroptosis in LPS + Nig-stimulated macrophages. Excessive pyroptosis induces inflammatory responses such as sepsis, as reported in numerous studies [13, 57], which reflects the deleterious side of pyroptosis. However, the occurrence of pyroptosis also has application value, which is reflected in the fact that pyroptosis can act an important role in anti-infection immunity [58] and anti-tumor immunity [59]. In fact, the current study may also provide a partial explanation that why DHEA has the ability to resist foreign microbial infection [60–63] while it also has anti-inflammatory function [19, 21, 64]. Of course, the fine regulation mechanism of DHEA on the pyroptosis is still a research worthy of further exploration.

In conclusion, our data indicated DHEA can inhibit the inflammatory signal and NLRP3 inflammasome activation in inflammatory macrophages. However, DHEA can also promote p62 puncta formation by activating ERK signal and inducing abnormal autophagy in a GPER-dependent manner, and which finally activates the caspase-8-GSDMD alternate pathway to exacerbate Nig-induced pyroptosis in LPS-primed macrophages. Meanwhile, the over-accumulated ROS further promotes the cell death in inflammatory macrophages (Fig. 6E). The regulation of pyroptosis by DHEA may contribute to its application in anti-infection immunity.

## Materials and methods

### Reagents and antibodies

DHEA, dimethyl sulfoxide (DMSO), and LPS (*Escherichia coli* 055:B5) were provided by Sigma (St Louis, MO, USA). The fetal bovine serum (FBS) was obtained from Gibco (Erie, NY, USA). The Dulbecco's modified Eagle’s medium (DMEM) and trypsin-EDTA were obtained from Biological Industries (Kibbutz Beit-Haemek, Israel). Shanghai Hengyuan Biological Technology Co., Ltd. (Shanghai, China) provided the commercial mouse TNF-α and IL-1β ELISA kit. The MCE (St. Louis, MO, USA) provided the Nigericin and inhibitors that included G1, G15, MCC950, BAY11-7082, rapamycin, and compound C. The lactate dehydrogenase (LDH) activity and reactive oxygen species detection kit were provided by Beyotime Biotechnology Institute (Shanghai, China).

Rabbit anti-p65 (#8242), ERK (#4695), phospho-ERK (#4370), cleaved caspase-8 (#8592), and phospho-AMPK (#2535) antibodies were purchased from Cell Signaling Technology (Boston, MA, USA). Bioword (Nanjing, China) provided the rabbit anti-NLRP3 (BS90949), p62 (AP6006), LAMP1 (BS6978) antibodies, and goat anti-mouse IgG (H + L) HRP (BS12478). Santa Cruze (Santa Cruz, CA, USA) provided the mouse anti-phospho-mTOR (sc-293133), mTOR (sc-517464) and LC3 (sc-398822) antibodies. Rabbit anti-IL-1β (A1112), caspase-1 (A16792) and GSDMD (A20197) antibodies were obtained from ABclonal (Wuhan, China). Rabbit anti-cleaved N-terminal GSDMD (GSDMD-NT) (ab215203) antibody was obtained from Abcam (Cambridge, UK). Rabbit anti-ASC (WL02462) antibody was purchased from Wanleibio (Shenyang, China). Proteintech Group (Rosemont, IL, USA) provided the rabbit anti-IkBα (10268-1-AP) antibody and HRP-conjugated Affinipure Goat Anti-Rabbit IgG (H + L) (SA00001-2). Biosharp (Hefei, China) provided the goat Anti-Rabbit IgG (H + L) FITC (BL033A) antibody.

### Cell culture and cell viability assay

Murine J774A.1 and RAW264.7 macrophages were grown in the DMEM culture medium containing 10% fetal calf serum at 37 °C humidified incubator with 5% CO_2_. The effects of DHEA on the viability of J774A.1 cells were determined by CCK-8 assay. Briefly, cells were seeded in 96-well tissue culture plates (1 × 10^4^ cells/well), and then treated with different doses of DHEA for 12 h. After that, 10 μL of CCK-8 was added into each well and incubated for 1 h. The OD_450_ values were measured using a model 550 Microplate reader.

### Cell stimulation

Macrophages were seeded in 6-well tissue culture plates (1.2 × 10^6^ cells/well) or 24-well tissue culture plates (2.5 × 10^5^ cells/well), and were pre-treated with different doses of DHEA (0, 10, 20, 50 μM) for 1 h. Then, the cells were primed with 100 ng/mL LPS for 4 h, follow stimulated with NLRP3 activators Nig (10 μM) or ATP (2 mM) for another 1 h.

### Microscopy imaging for cell death

After indicated treatments for stimulation, the cells were stained with propidium iodide (PI; 1 µg/mL) according to manufacturer’s instruction, and the Hochest 33342 was used to stain the cell nuclei. A fluorescence microscope was used to take bright-field and fluorescence images. PI-positive dead cells in 5 randomly selected fluorescence microscope images were counted by Image J software.

### LDH determination for cell death

After indicated treatments for stimulation, the cell death was measured by detecting the lactate dehydrogenase (LDH) release (%). The LDH release in cell culture supernatant was measured according to the manufacturer’s instruction.

### DCFH-DA and LysoTracker Red staining

The intracellular ROS and lysosomal rupture were detected using DCFH-DA (Beyotime) and LysoTracker Red (Beyotime), respectively. Briefly, after indicated treatments for stimulation, the cells were added with DCFH-DA (10 μM) or LysoTracker Red (100 nM) for 30 min. After that, the fluorescence intensity was immediately detected using a fluorescence microscope.

### Western blotting

The total proteins were extracted according to the manufacturer’s protocol. The concentrations of proteins were determined by the Pierce BCA protein assay kit. The protein expression levels were detected using western blot according the previous reported [20]. β-Tubulin was used for internal reference and the Image J software was used to quantify blots. All full and uncropped western blots are uploaded as ‘Supplementary Material—Original western blots’.

### Immunofluorescence (IF)

The immunofluorescence analysis was performed as previously described [20]. Briefly, cells were fixed with 4% paraformaldehyde for 10 min and washed with PBS; then the cells were incubated with 0.1% Triton X-100 for 30 min. After that, cells were blocked with 5% BSA and incubated with the primary antibodies (LC3, 1:100 dilution; p62, 1:100 dilution; LAMP1, 1:100 dilution; mTOR, 1:100 dilution; cleaved caspase-8, 1:100 dilution; GSDMD-NT, 1:100 dilution; ASC, 1:100 dilution) followed by incubation with the fluorescently labeled secondary antibodies. The cells were dyed with DAPI and analyzed by the laser scanning confocal microscope.

### ELISA

The TNF-α and IL-1β concentrations in cell culture supernatant were detected using the commercial ELISA kits according to the instructions.

### Statistical analysis

The data were expressed as mean ± SEM, one-way ANOVA and Student–Newman–Keuls test were performed by Prism 8.0.2 software for comparing the significant differences among different groups.

## Supplementary information

Reproducibility checklist

Supplemental Information

Original western blots

Cover Art

## Data Availability

The data supporting the conclusions of this article are included within the published article and its supplementary information files.
