# Supplementary material for: Dehydroepiandrosterone exacerbates nigericin-induced abnormal autophagy and pyroptosis via GPER activation in LPS-primed macrophages
Source: Cell Death Dis. 2022 Apr 19;13(4):372. doi: 10.1038/s41419-022-04841-6 (PMC9018772; doi:10.1038/s41419-022-04841-6)
Supplement: Supplementary file 2 — Supplemental Information [file 41419_2022_4841_MOESM2_ESM.docx]

**Supplemental Information**

**Dehydroepiandrosterone exacerbates nigericin-induced abnormal autophagy and pyroptosis via GPER activation in LPS-primed macrophages**

**Ji Cao, Longlong Li, Yao Yao, Yuxiao Xing, Haitian Ma***

**S Figure 1**

**S Figure 1.** DHEA promotes Nig-induced pyroptosis in LPS-primed macrophages. Related to **Figure 2**

(A) J774A.1 cells were treated with DHEA (0, 10, 20, 50 or 100 μM) for 12 h, and the cell viabilities were detected by CCK-8 assay, n=6. (B-C) Cells were pre-treated with DHEA (20 μM) in the presence or absence of MCC950 (10 μM) or BAY11-7082 (10 μM) for 1 h, and primed with LPS for 4 h, then stimulated with nigericin (Nig) for 1 h, PI-positive dead cells in 5 randomly selected fluorescence microscope images were counted by ImageJ software, scale bar = 200 μm. (D) TNF-α content in cell culture supernatant, n=4. Data are presented as means ± SEM. **P* < 0.05, ***P* < 0.01, compared with the respective control.

**S Figure 2**

**S Figure 2.** DHEA induces abnormal autophagy and excessive ROS release in Nig-treated inflammatory macrophages. Related to **Figure 3**

(A) J774A.1 cells were pre-treated with different doses of DHEA (50 μM) for 1 h and primed with 100 ng/mL LPS for 4 h; then stimulated with NLRP3 activators nigericin (10 μM) for 1 h. The autophagic flux were analyzed by immunofluorescence through using the mCherry-GFP-LC3B adenovirus reporter, scale bar = 20 μm.

**
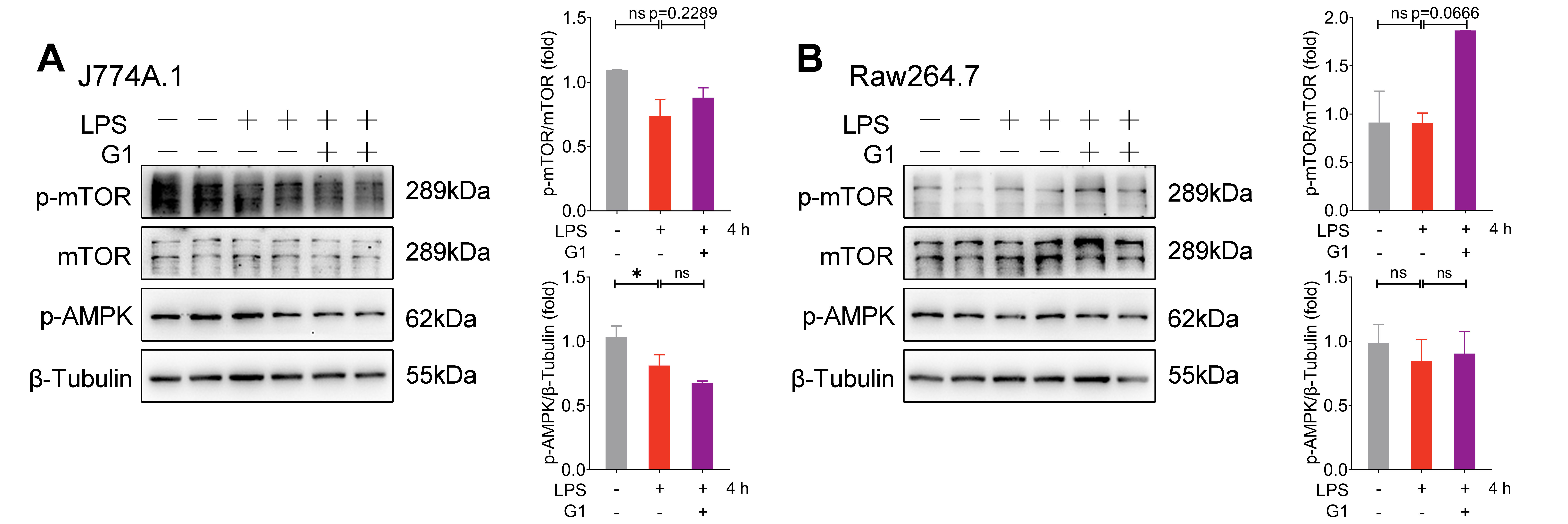
S Figure 3**

**S Figure 3.** GPER agonist G1 has no influence on AMPK/mTOR signal in inflammatory macrophages. Related to **Figure 4**

(A-B) J774A.1 and RAW264.7 cells were pre-treated with G1 (1 μM) for 1 h, then stimulated with LPS for 4 h, the p-mTOR and p-AMPK protein expression levels were measured by western blotting and quantified by Image J software. Data are presented as means ± SEM (n=4). **P* < 0.05, ***P* < 0.01, compared with the respective control.

**
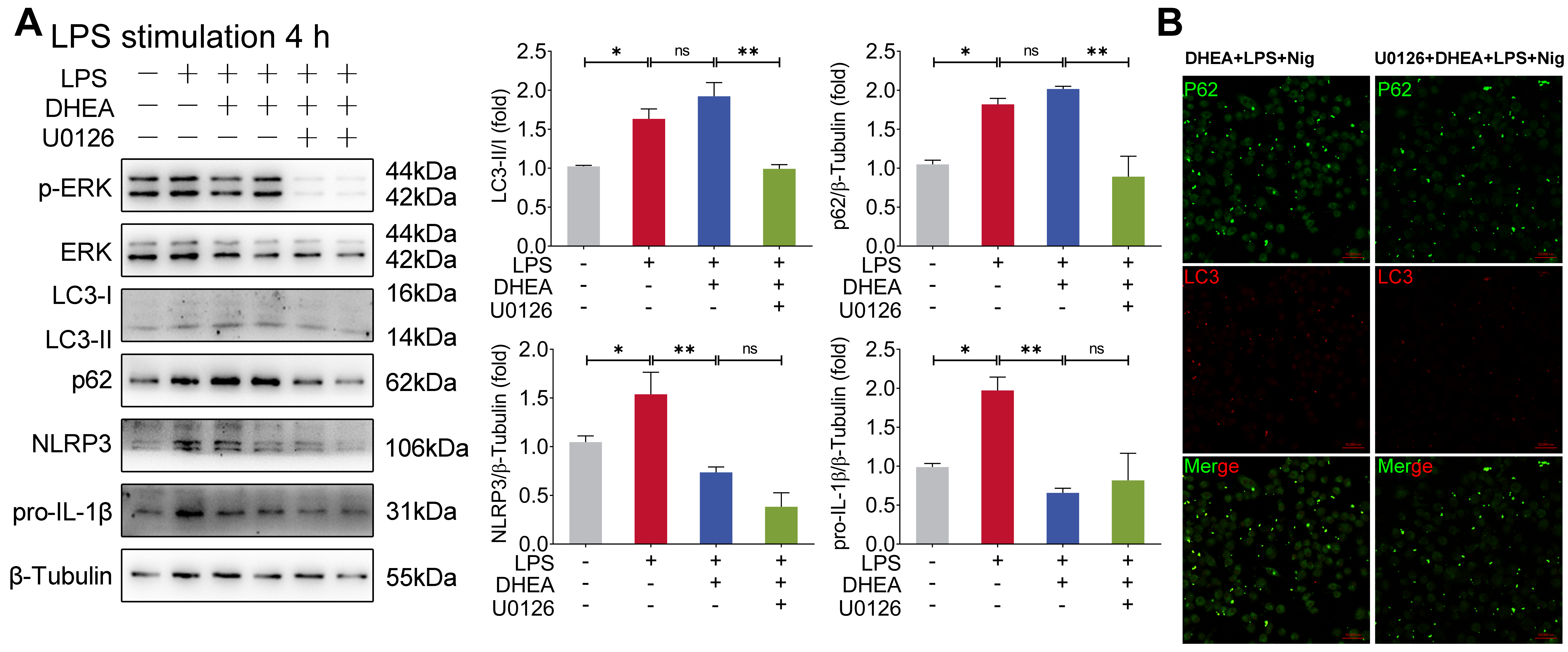
S Figure 4**

**S Figure 4.** ERK signaling is upstream of p62. Related to **Figure 5**

(A) J774A.1 cells were pre-treated with DHEA (50 μM) in the presence or absence of the ERK inhibitor U0126 (10 μM) for 1 h, then stimulated with LPS for 4 h, the indicated protein expression levels were measured by western blotting and quantified by Image J software. (B) Cells were pre-treated with DHEA (20 μM) in the presence or absence of U0126 for 1 h, and primed with LPS for 4 h; then stimulated with nigericin (Nig) for 1 h, the formation of p62/LC3 puncta was analyzed by immunofluorescence, scale bar = 50 μm. Data are presented as means ± SEM (n=3 or 4). **P* < 0.05, ***P* < 0.01, compared with the respective control.

**
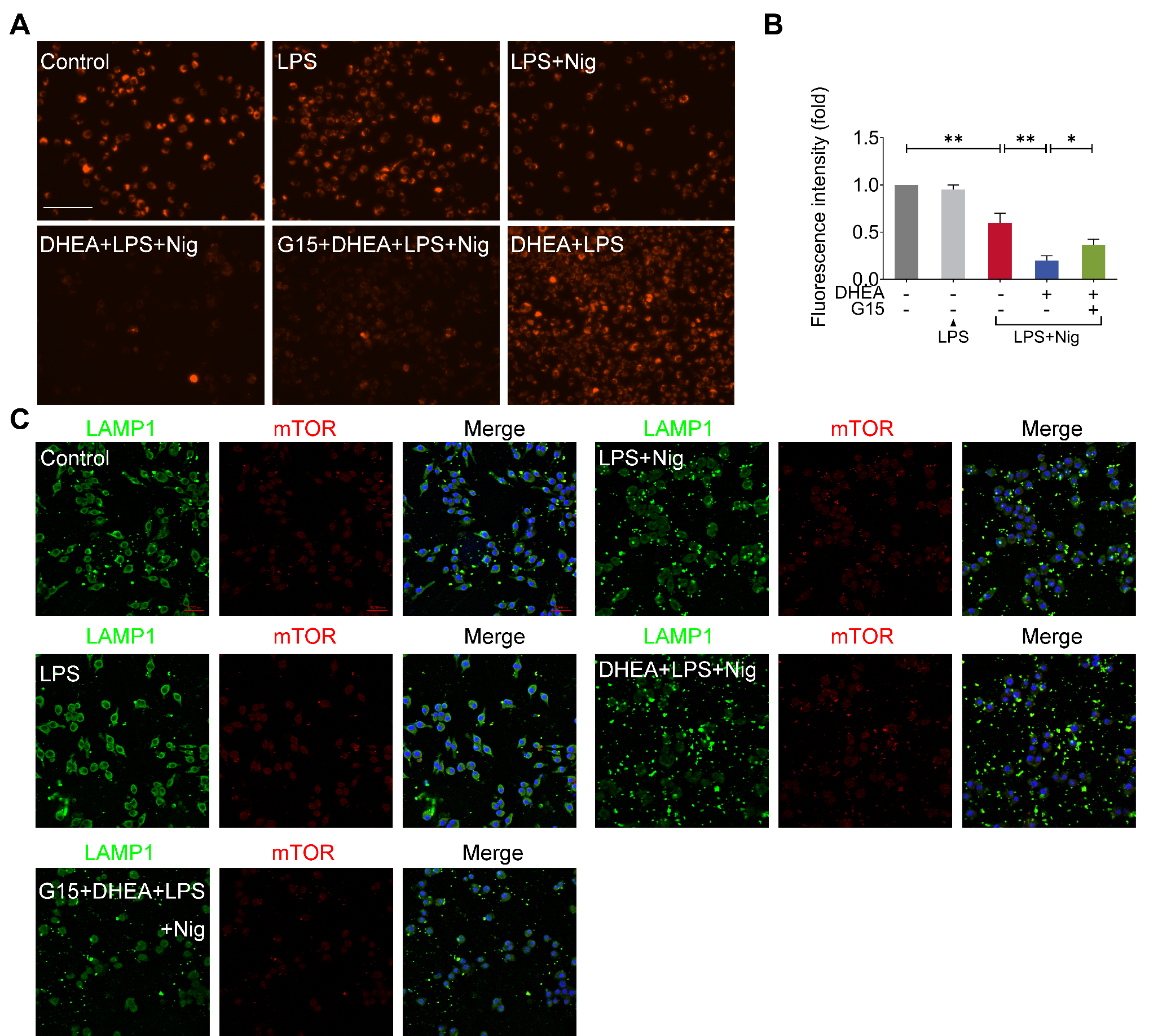
S Figure 5**

**S Figure 5.** DHEA promotes Nig-induced lysosomal damage in LPS-primed macrophages via GPER activation. Related to **Figure 5**

(A-B) J774A.1 cells were pre-treated with DHEA (50 μM) in the presence or absence of the GPER inhibitor G15 (1 μM) for 1 h, then stimulated with LPS for 4 h. The lysosomal damage was detected using LysoTracker Red staining, scale bar = 100 μm. (C) The LAMP1/mTOR overlay was analyzed by immunofluorescence, scale bar = 50 μm. Data are presented as means ± SEM (n=3). **P* < 0.05, ***P* < 0.01, compared with the respective control.

**
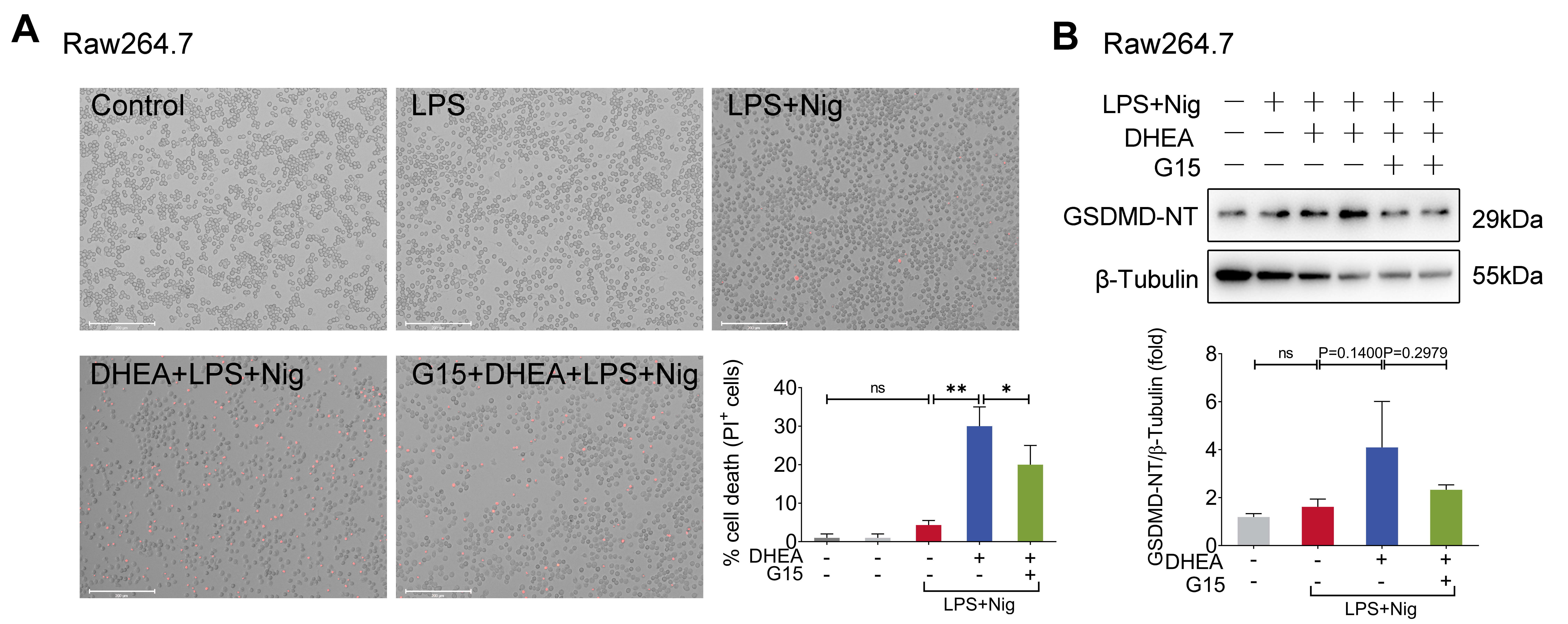
S Figure 6**

**S Figure 6.** DHEA exacerbates Nig-induced pyroptosis in LPS-primed macrophages via GPER activation. Related to **Figure 6**

(A) RAW264.7 cells were pre-treated with DHEA (50 μM) in the presence or absence of the GPER inhibitor G15 (1 μM) for 1 h, then stimulated with LPS for 4 h, PI-positive dead cells were counted by ImageJ software, scale bar = 200 μm. (B) The GSDMD-NT protein expression levels were measured by western blotting and quantified by Image J software. Data are presented as means ± SEM (n=3 or 4). **P* < 0.05, ***P* < 0.01, compared with the respective control.

**
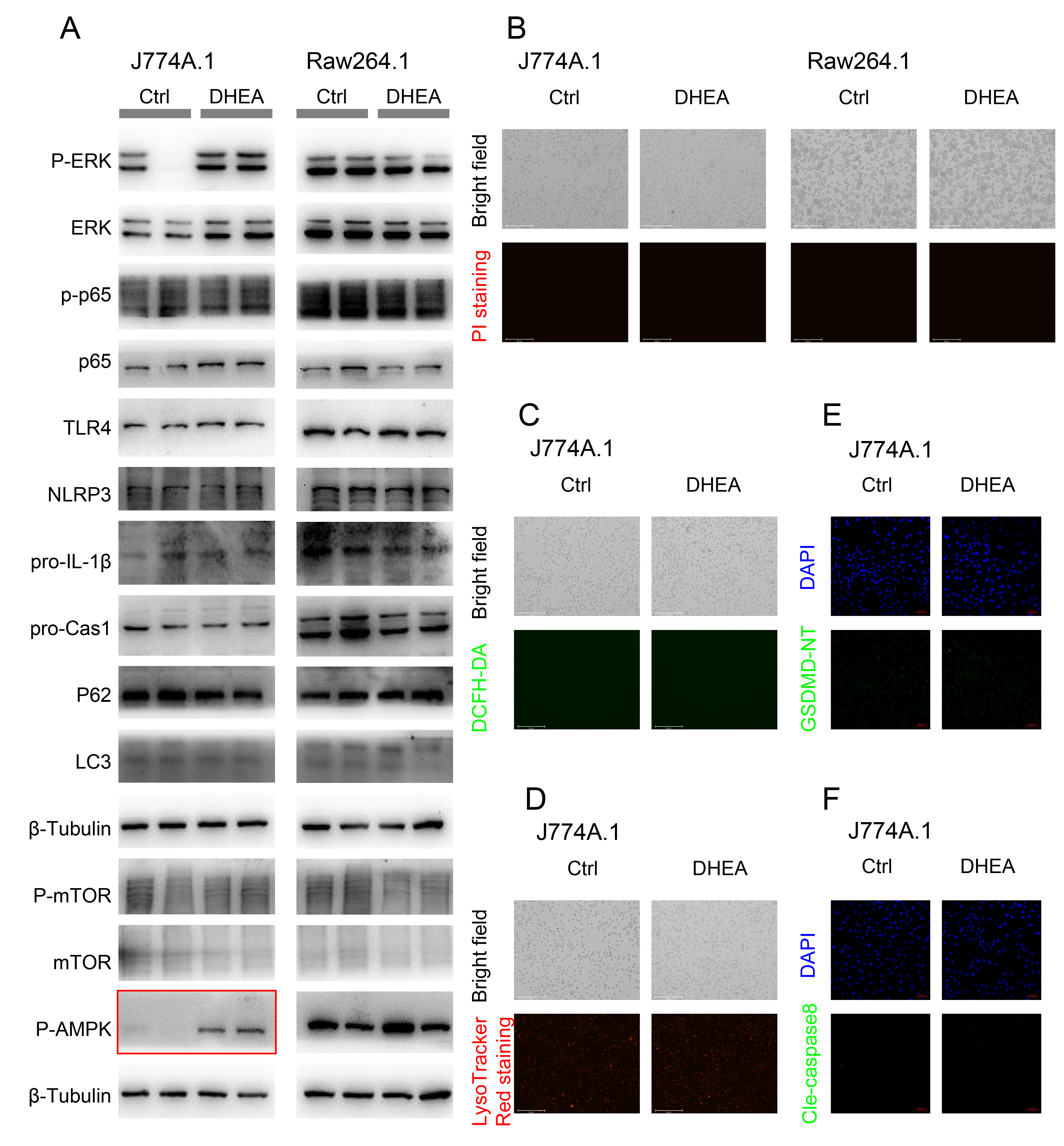
S Figure 7**

**S Figure 7.** Effects of DHEA alone treatment on the indicated signaling pathways and phenotypes in macrophages.

(A) J774A.1 and RAW264.7 cells were treated with or without DHEA (50 μM) for 5 h, and the indicated protein expression levels were measured by western blotting. (B) J774A.1 and RAW264.7 cells were treated with or without DHEA (50 μM) for 5 h. The cell death was detected using PI staining, scale bar = 200 μm. (C) The intracellular ROS levels were detected using DCFH-DA, scale bar = 200 μm. (D) The lysosomal function was detected using LysoTracker Red staining, scale bar = 200 μm. (E-F) The GSDMD-NT and Cle-caspase8 protein levels were analyzed by immunofluorescence, scale bar = 50 μm.
