# Supplementary material for: Dehydroepiandrosterone exacerbates nigericin-induced abnormal autophagy and pyroptosis via GPER activation in LPS-primed macrophages
Source: Cell Death Dis. 2022 Apr 19;13(4):372. doi: 10.1038/s41419-022-04841-6 (PMC9018772; doi:10.1038/s41419-022-04841-6)
Supplement: Supplementary file 3 — Original western blots [file 41419_2022_4841_MOESM3_ESM.ppt]

## Slide 1
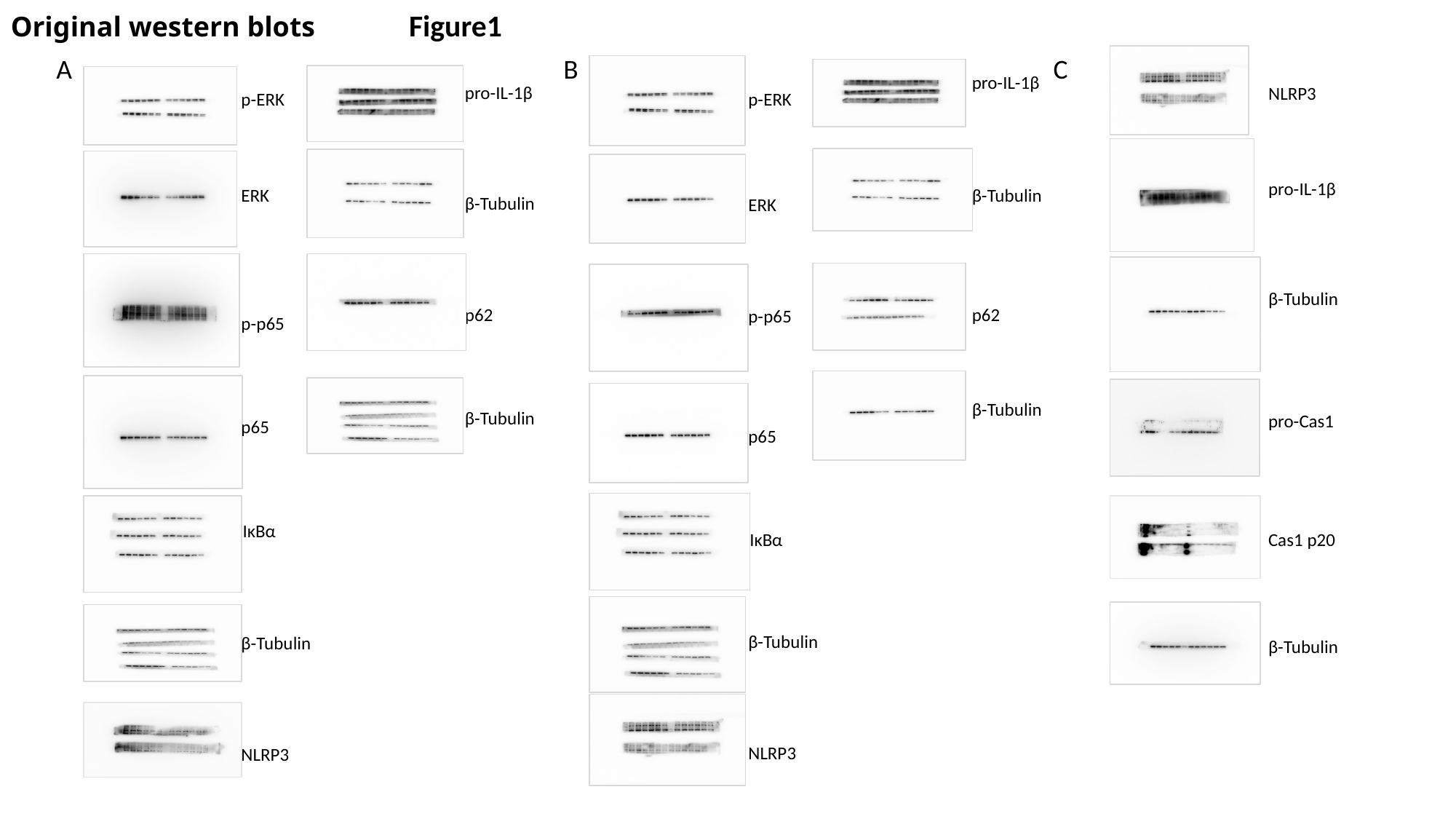

Figure1
Original western blots
A
B
C
pro-IL-1β
pro-IL-1β
NLRP3
p-ERK
p-ERK
pro-IL-1β
ERK
β-Tubulin
β-Tubulin
ERK
β-Tubulin
p62
p62
p-p65
p-p65
β-Tubulin
β-Tubulin
pro-Cas1
p65
p65
IκBα
IκBα
Cas1 p20
β-Tubulin
β-Tubulin
β-Tubulin
NLRP3
NLRP3

## Slide 2
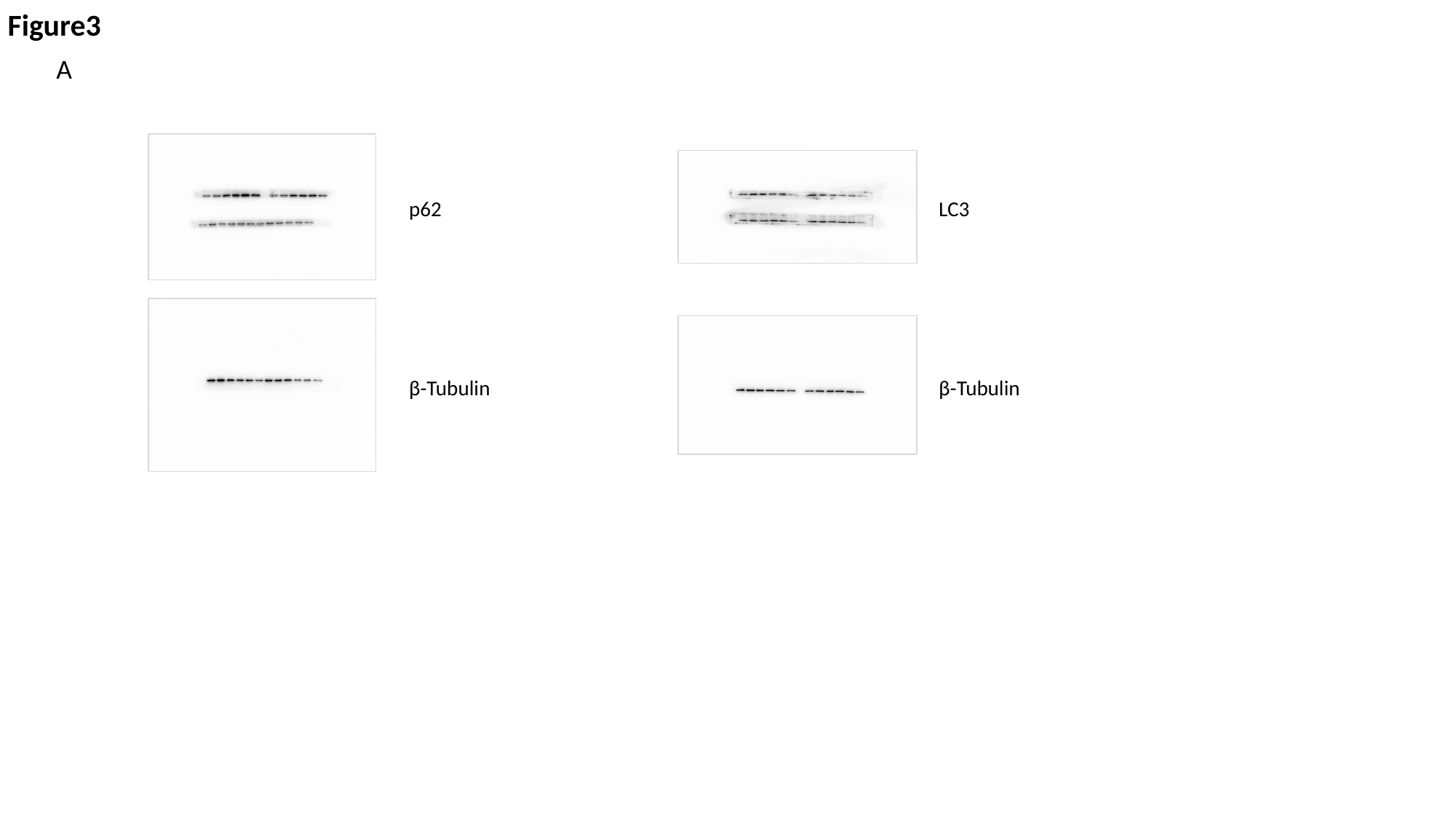

Figure3
A
p62
LC3
β-Tubulin
β-Tubulin

## Slide 3
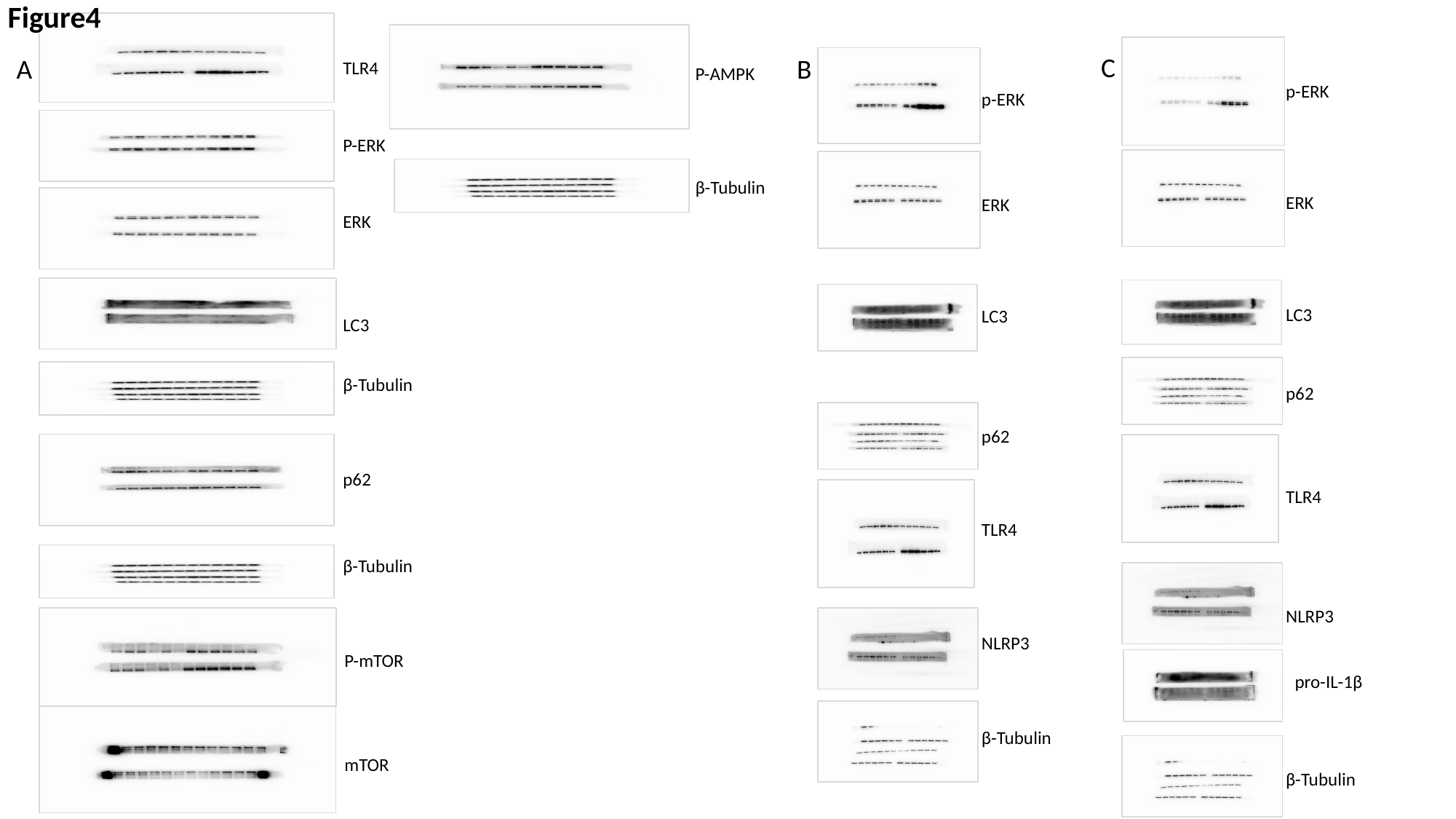

Figure4
C
A
B
TLR4
P-AMPK
p-ERK
p-ERK
P-ERK
β-Tubulin
ERK
ERK
ERK
LC3
LC3
LC3
β-Tubulin
p62
p62
p62
TLR4
TLR4
β-Tubulin
NLRP3
NLRP3
P-mTOR
pro-IL-1β
β-Tubulin
mTOR
β-Tubulin

## Slide 4
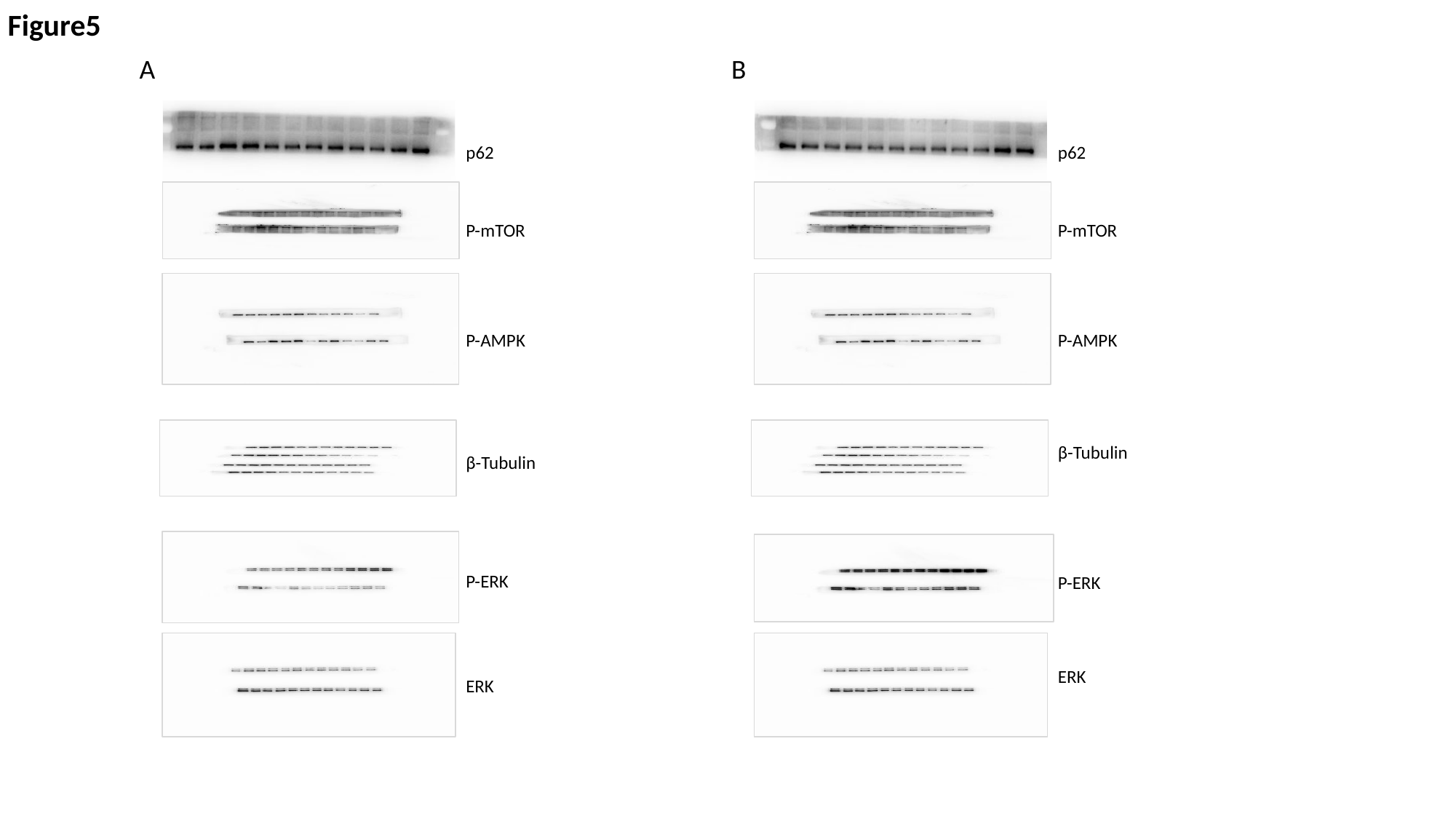

Figure5
A
B
p62
p62
P-mTOR
P-mTOR
P-AMPK
P-AMPK
β-Tubulin
β-Tubulin
P-ERK
P-ERK
ERK
ERK

## Slide 5
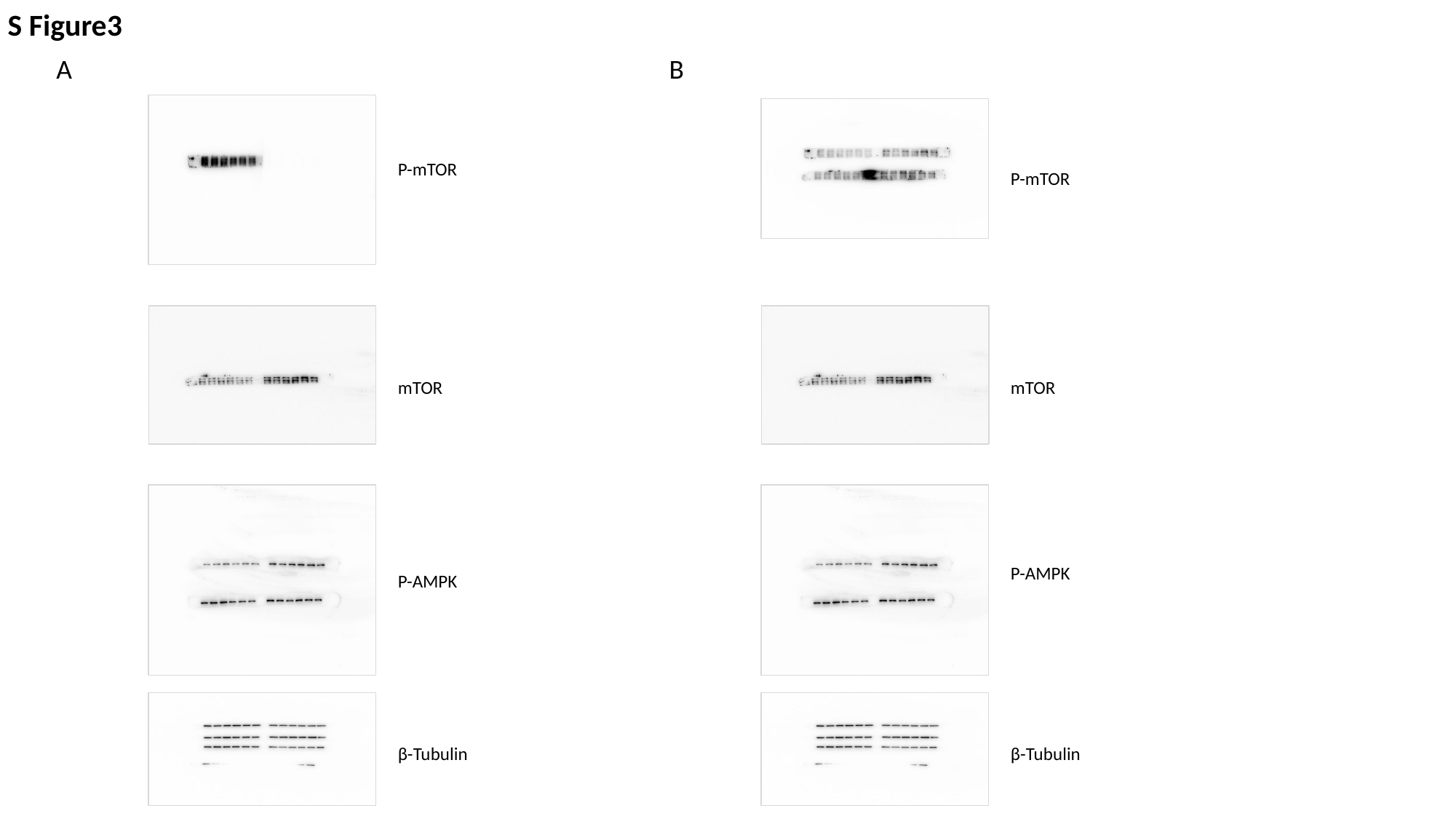

S Figure3
A
B
P-mTOR
P-mTOR
mTOR
mTOR
P-AMPK
P-AMPK
β-Tubulin
β-Tubulin

## Slide 6
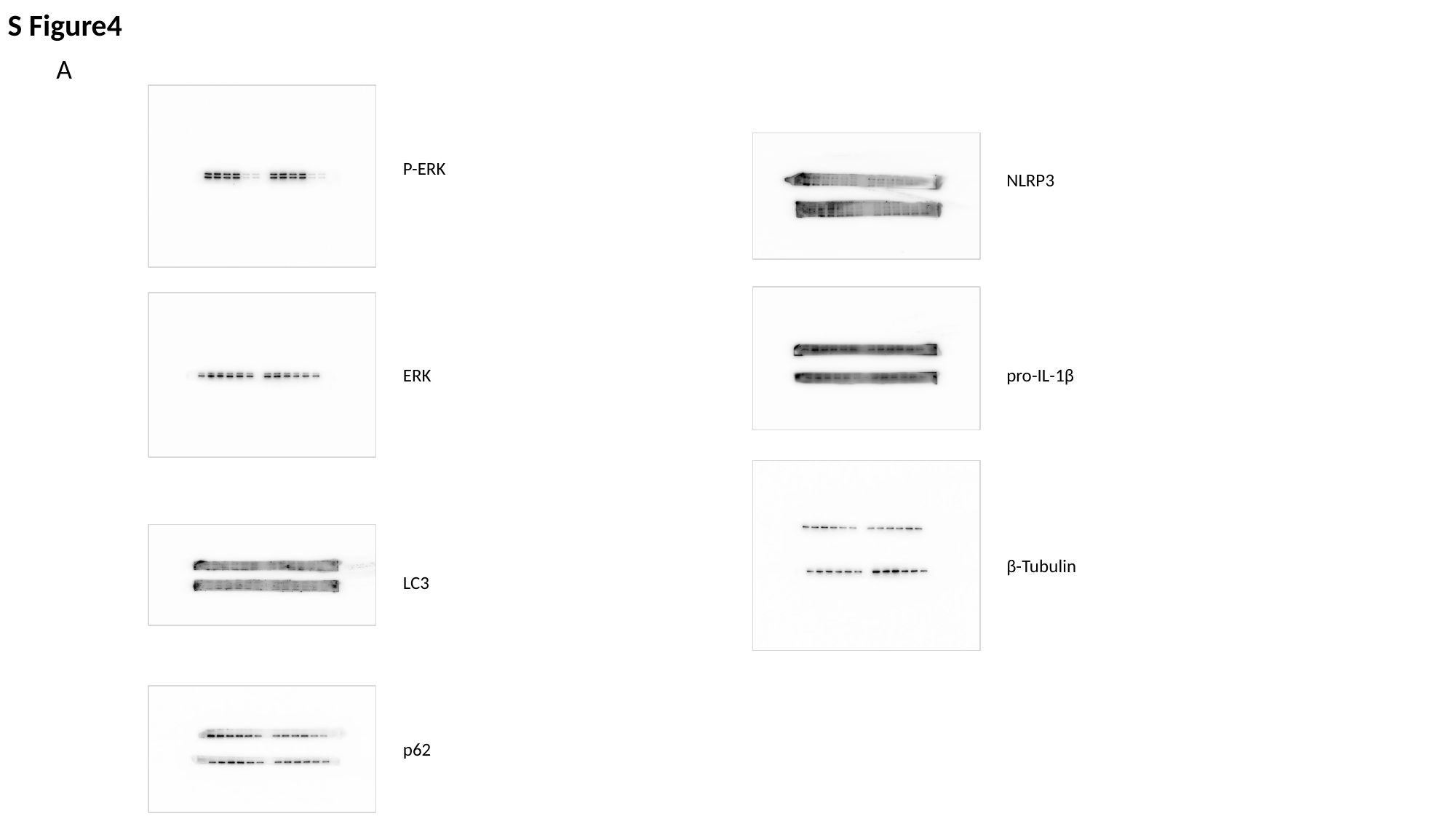

S Figure4
A
P-ERK
NLRP3
ERK
pro-IL-1β
β-Tubulin
LC3
p62

## Slide 7
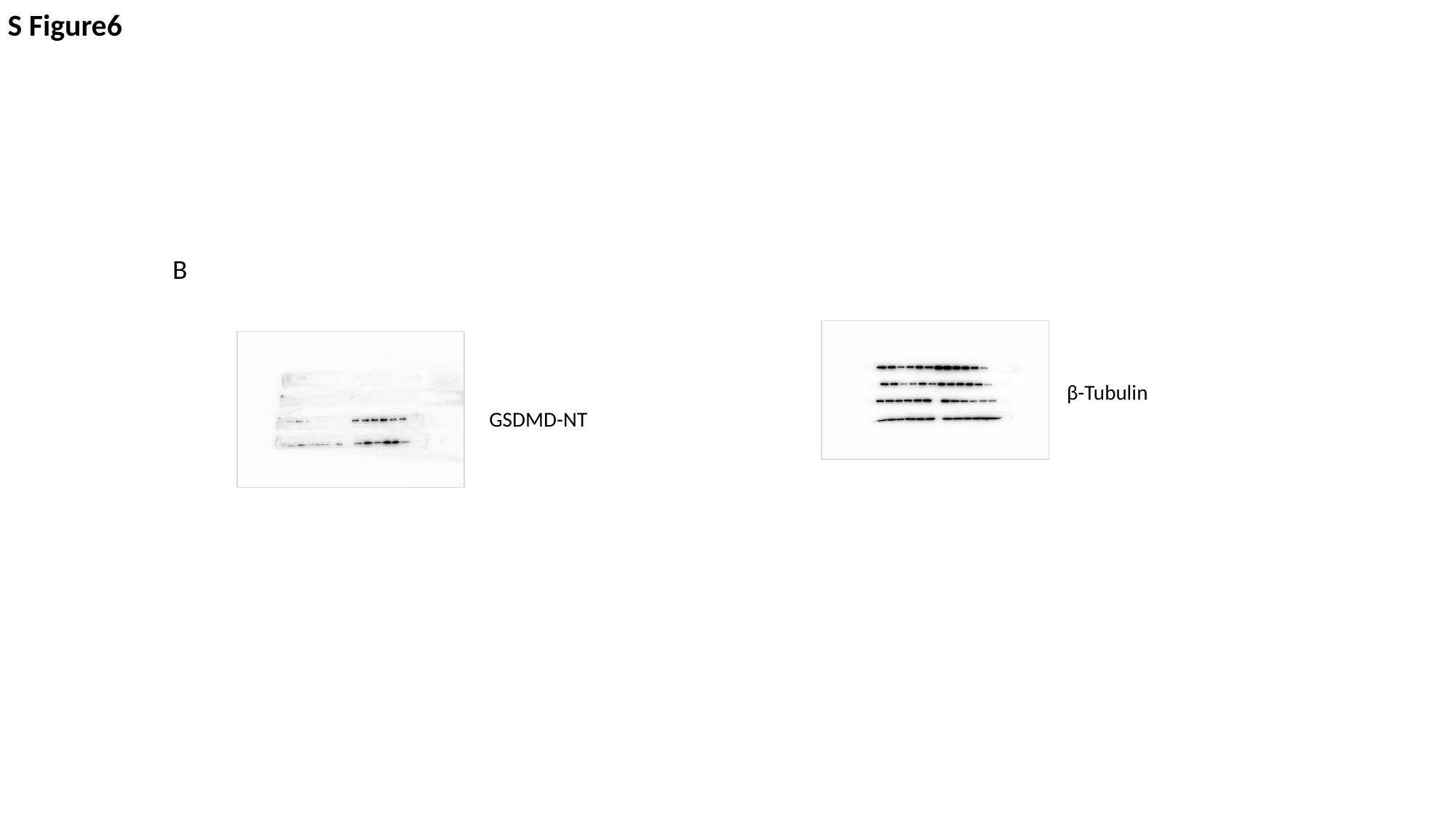

S Figure6
B
β-Tubulin
GSDMD-NT

## Slide 8
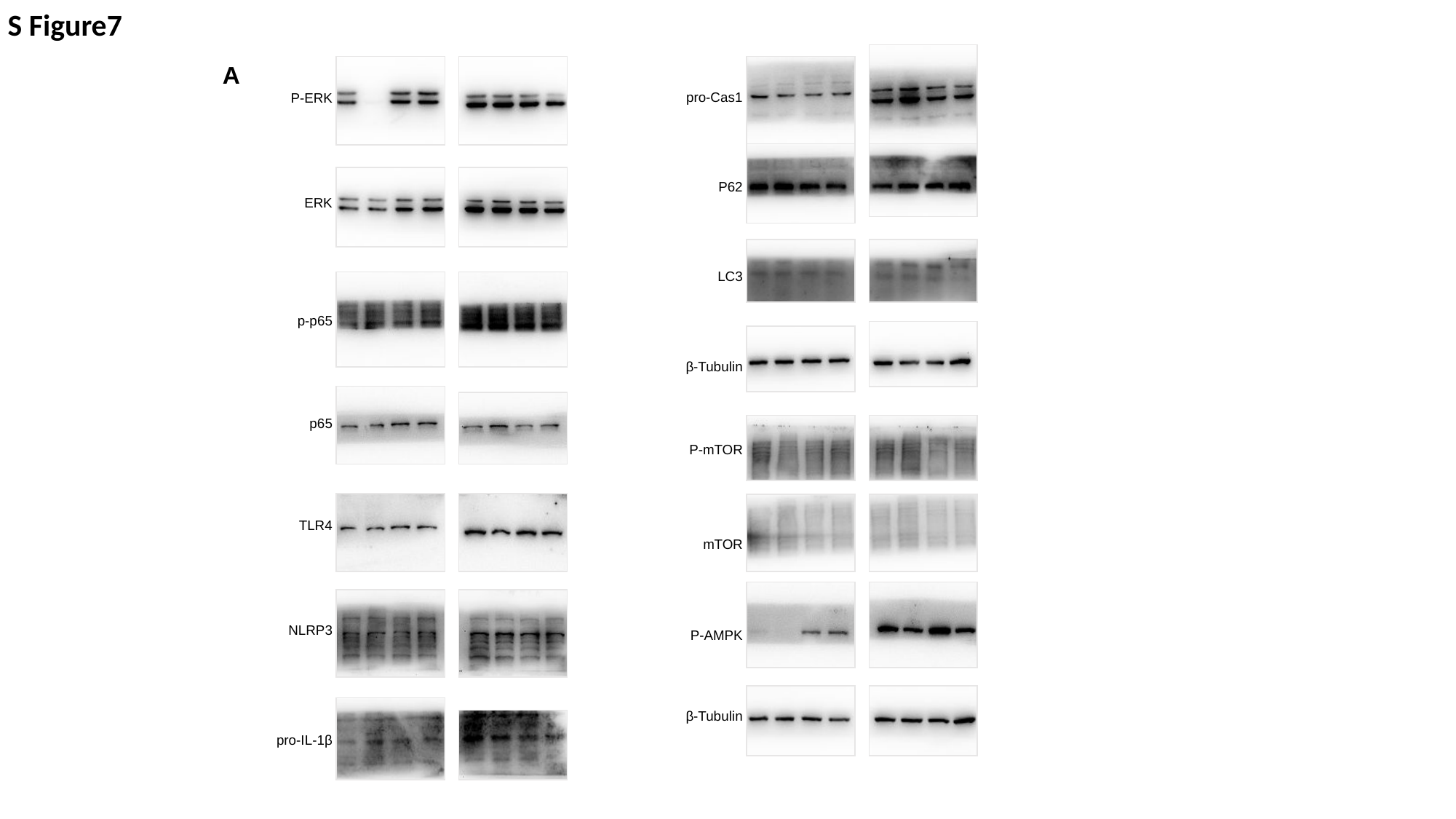

S Figure7
A
pro-Cas1
P-ERK
P62
ERK
LC3
p-p65
β-Tubulin
p65
P-mTOR
TLR4
mTOR
NLRP3
P-AMPK
β-Tubulin
pro-IL-1β
