# Supplementary figures and images for: Dehydroepiandrosterone exacerbates nigericin-induced abnormal autophagy and pyroptosis via GPER activation in LPS-primed macrophages
Source: Cell Death Dis. 2022 Apr 19;13(4):372. doi: 10.1038/s41419-022-04841-6 (PMC9018772; doi:10.1038/s41419-022-04841-6)

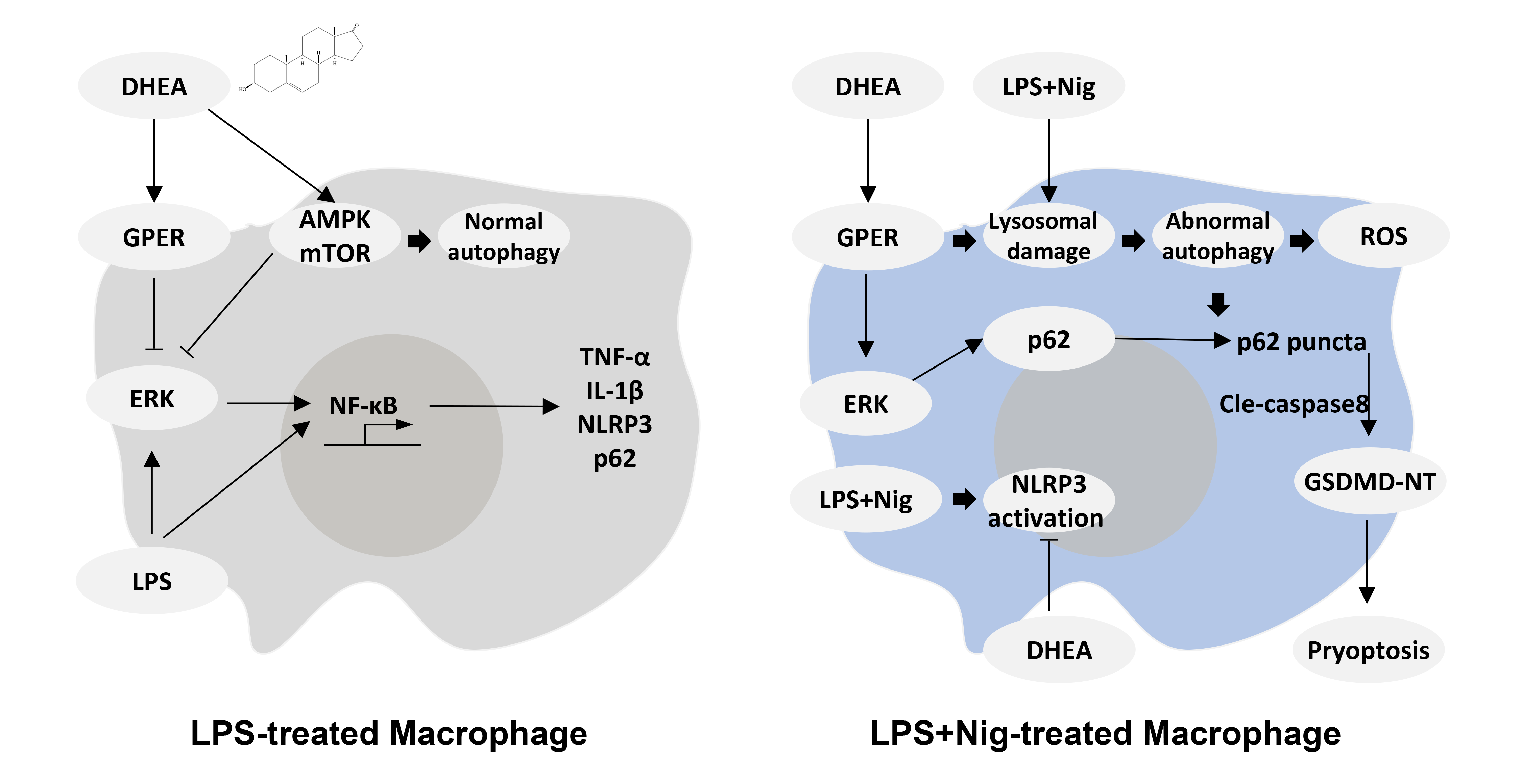

Supplement: Supplementary file 4 — Cover Art [file 41419_2022_4841_MOESM4_ESM.tif]
